# Supplementary material for: A Likelihood Approach for Real-Time Calibration of Stochastic Compartmental Epidemic Models
Source: PLoS Comput Biol. 2017 Jan 17;13(1):e1005257. doi: 10.1371/journal.pcbi.1005257 (PMC5240920; doi:10.1371/journal.pcbi.1005257)
Supplement: S1 File — (TAR.GZ) [file pcbi.1005257.s014.tar.gz › HSPH_Online-SI-Revision/Additions/IPoi10/n100-extreme-SEITR_noisy/n100-extreme-SEITR_noisy_BENCH2_table-8.pdf]

1	newly 3 weeks spec	2107.88	1613.2	{1028., 2381.}	32.1743	0.173936	newly 3 weeks cumul	3792.89	5300.33	{3619., 7205.}	45.7401	0.160493	newly 5 weeks spec	7087.2	1184.2	{343., 2017.}	83.291	0.848526	newly 5 weeks cumul	15 036.4	7911.8	{5287., 10 875.}	47.3825	0.294039	newly 58 weeks cumul	94 514.	12 613.7	{7637., 18 914.}	86.6542	0.917217
2	newly 3 weeks spec	265.	1.31579	{0., 3.}	99.5035	2.71146	newly 3 weeks cumul	343.	3.7193	{0., 8.}	98.9157	2.28109	newly 5 weeks spec	3034.	2.19298	{0., 6.}	99.9277	3.68391	newly 5 weeks cumul	4334.	7.68421	{0., 17.}	99.8227	3.14007	newly 66 weeks cumul	99 017.	20 286.4	{0., 66 651.}	79.5122	2.71512
3	newly 3 weeks spec	257.	1.7	{0., 6.}	99.3385	2.81895	newly 3 weeks cumul	379.	4.64	{0., 15.}	98.7757	2.60181	newly 5 weeks spec	2684.	3.83	{0., 8.}	99.8573	3.77579	newly 5 weeks cumul	3894.	11.08	{0., 33.}	99.7155	3.45784	newly 68 weeks cumul	98 993.	17 020.8	{0., 71 619.}	82.8061	3.74265
4	newly 3 weeks spec	698.	6.69643	{0., 22.}	99.0406	2.79695	newly 3 weeks cumul	1030.	14.75	{0., 43.}	98.568	2.48406	newly 5 weeks spec	3933.	17.6607	{0., 49.}	99.551	3.42507	newly 5 weeks cumul	6733.	43.5536	{0., 127.}	99.3531	3.05031	newly 66 weeks cumul	96 862.	25 345.4	{0., 75 304.}	73.8335	2.67296
5	newly 3 weeks spec	187.	1.73	{0., 6.}	99.0749	2.73924	newly 3 weeks cumul	237.	4.1	{0., 13.}	98.27	2.48739	newly 5 weeks spec	3231.	5.6	{0., 6.}	99.8267	3.93779	newly 5 weeks cumul	4188.	12.57	{0., 26.}	99.6999	3.60109	newly 68 weeks cumul	98 880.	12 878.5	{0., 78 221.}	86.9757	4.33827
6	newly 3 weeks spec	323.	2.61644	{0., 6.}	99.19	2.71177	newly 3 weeks cumul	460.	5.32877	{0., 10.}	98.8416	2.44155	newly 5 weeks spec	3690.	7.0137	{0., 21.}	99.8099	3.66058	newly 5 weeks cumul	5281.	17.0959	{0., 38.}	99.6763	3.2265	newly 67 weeks cumul	95 041.	26 088.5	{0., 78 838.}	72.5502	2.94499
7	newly 3 weeks spec	2336.	1770.26	{177., 4002.}	61.0748	0.506731	newly 3 weeks cumul	4235.	3543.21	{337., 8140.}	66.1947	0.509296	newly 5 weeks spec	6848.	3741.52	{566., 7343.}	49.5337	0.47985	newly 5 weeks cumul	15 415.	9940.05	{1248., 21 044.}	53.8151	0.491757	newly 59 weeks cumul	97 367.	83 979.4	{82 762., 84 817.}	13.7497	0.0642696
8	newly 3 weeks spec	476.	2.31746	{0., 5.}	99.5131	2.87215	newly 3 weeks cumul	697.	5.79365	{0., 11.}	99.1688	2.43333	newly 5 weeks spec	4729.	4.19048	{0., 10.}	99.9114	3.83332	newly 5 weeks cumul	7052.	12.9841	{0., 30.}	99.8159	3.22017	newly 66 weeks cumul	99 432.	22 224.	{1., 75 266.}	77.6491	2.67888
9	newly 3 weeks spec	88.	1.8	{0., 6.}	97.9545	2.38882	newly 3 weeks cumul	112.	3.91	{0., 10.}	96.5089	2.14153	newly 5 weeks spec	2227.	5.9	{0., 11.}	99.7351	3.79164	newly 5 weeks cumul	2748.	13.01	{0., 24.}	99.5266	3.3816	newly 68 weeks cumul	99 354.	14 857.2	{0., 75 191.}	85.0462	4.17483
10	newly 3 weeks spec	982.	137.51	{4., 313.}	86.0129	1.40187	newly 3 weeks cumul	1566.	258.039	{6., 556.}	84.6743	1.27727	newly 5 weeks spec	4646.	445.137	{8., 1063.}	90.4189	1.60499	newly 5 weeks cumul	8317.	961.706	{18., 2208.}	88.4369	1.48639	newly 64 weeks cumul	99 174.	72 515.1	{15 133., 92 373.}	26.881	0.371946
11	newly 3 weeks spec	578.	3.91892	{0., 15.}	99.322	2.6855	newly 3 weeks cumul	848.	11.0811	{0., 29.}	98.6933	2.35793	newly 5 weeks spec	4035.	5.24324	{0., 19.}	99.8701	3.51258	newly 5 weeks cumul	6425.	20.4595	{0., 57.}	99.6816	2.98769	newly 63 weeks cumul	99 600.	13 971.1	{0., 33 227.}	85.9728	2.33287
12	newly 3 weeks spec	936.	3.45714	{0., 9.}	99.6306	2.9776	newly 3 weeks cumul	1372.	8.62857	{1., 22.}	99.3711	2.51593	newly 5 weeks spec	5911.	5.28571	{0., 14.}	99.9106	3.50088	newly 5 weeks cumul	9781.	18.3429	{1., 44.}	99.8125	3.01824	newly 64 weeks cumul	98 924.	24 285.9	{1., 66 923.}	75.45	1.99349
13	newly 3 weeks spec	632.	4.6383	{0., 17.}	99.2661	2.91054	newly 3 weeks cumul	864.	12.1277	{0., 49.}	98.5963	2.57521	newly 5 weeks spec	4789.	9.74468	{0., 30.}	99.7965	3.77133	newly 5 weeks cumul	7468.	28.8511	{0., 91.}	99.6137	3.32414	newly 65 weeks cumul	99 507.	23 149.5	{0., 73 166.}	76.7358	3.06123
14	newly 3 weeks spec	1910.	1802.38	{429., 3880.}	59.5978	0.379478	newly 3 weeks cumul	3449.	3703.49	{785., 8034.}	67.2102	0.383697	newly 5 weeks spec	5421.	3659.7	{1085., 6705.}	42.9652	0.359476	newly 5 weeks cumul	12 064.	10 008.1	{2538., 20 018.}	50.974	0.374358	newly 58 weeks cumul	96 176.	86 168.	{85 544., 89 153.}	10.4059	0.135142
15	newly 3 weeks spec	1491.	673.241	{117., 1404.}	58.5227	0.575408	newly 3 weeks cumul	2425.	1382.39	{260., 2846.}	55.6159	0.513331	newly 5 weeks spec	6031.	1495.07	{307., 3026.}	75.2102	0.813364	newly 5 weeks cumul	11 560.	3891.85	{776., 7872.}	66.3335	0.69689	newly 59 weeks cumul	97 278.	76 277.3	{77 069., 78 161.}	21.5883	0.206591
16	newly 3 weeks spec	1486.	1702.58	{344., 3577.}	60.8208	0.284667	newly 3 weeks cumul	2628.	3120.46	{612., 6678.}	64.4139	0.289267	newly 5 weeks spec	4920.	4614.49	{1212., 7860.}	40.5943	0.22992	newly 5 weeks cumul	10 361.	10 653.8	{2461., 20 219.}	49.2857	0.254911	newly 62 weeks cumul	92 728.	91 828.2	{88 165., 92 976.}	1.06543	0.00475084
17	newly 3 weeks spec	187.	3.03	{0., 13.}	98.3797	2.55464	newly 3 weeks cumul	247.	6.77	{0., 24.}	97.2591	2.22036	newly 5 weeks spec	2188.	9.48	{0., 28.}	99.5667	3.5882	newly 5 weeks cumul	3077.	21.61	{0., 79.}	99.2977	3.11818	newly 68 weeks cumul	93 253.	16 985.	{0., 75 437.}	81.7861	3.5843
18	newly 3 weeks spec	162.	2.2	{0., 6.}	98.642	2.51207	newly 3 weeks cumul	222.	5.07143	{0., 15.}	97.7156	2.20155	newly 5 weeks spec	2807.	4.88571	{0., 13.}	99.8259	3.64834	newly 5 weeks cumul	3654.	13.4857	{0., 30.}	99.6309	3.14257	newly 67 weeks cumul	99 640.	26 131.1	{0., 78 565.}	73.7745	3.0211
19	newly 3 weeks spec	726.	4.63158	{0., 9.}	99.362	2.70713	newly 3 weeks cumul	1060.	11.7105	{1., 31.}	98.8952	2.33123	newly 5 weeks spec	4054.	6.02632	{0., 13.}	99.8513	3.43143	newly 5 weeks cumul	6766.	22.7895	{1., 58.}	99.6632	2.9212	newly 62 weeks cumul	99 318.	15 617.7	{1., 37 637.}	84.2751	2.28197
20	newly 3 weeks spec	300.	2.41429	{0., 7.}	99.1952	2.79415	newly 3 weeks cumul	402.	5.64286	{0., 14.}	98.5963	2.45017	newly 5 weeks spec	3692.	4.91429	{0., 18.}	99.8669	3.76977	newly 5 weeks cumul	5182.	13.5286	{0., 42.}	99.7389	3.29857	newly 67 weeks cumul	98 887.	22 534.2	{0., 74 744.}	77.2122	3.15005
21	newly 3 weeks spec	120.	1.37	{0., 3.}	98.8583	2.60711	newly 3 weeks cumul	157.	3.53	{0., 9.}	97.7516	2.29004	newly 5 weeks spec	2417.	4.48	{0., 6.}	99.8146	3.89764	newly 5 weeks cumul	3205.	10.35	{0., 18.}	99.6771	3.42353	newly 68 weeks cumul	99 034.	15 666.8	{0., 73 200.}	84.1804	3.97749
22	newly 3 weeks spec	1137.	919.524	{25., 2126.}	62.6726	0.619754	newly 3 weeks cumul	1829.	1593.71	{51., 3745.}	66.3343	0.601125	newly 5 weeks spec	5958.	3071.81	{137., 6447.}	55.4266	0.682311	newly 5 weeks cumul	10 630.	6409.67	{261., 14 025.}	55.9869	0.65955	newly 64 weeks cumul	96 878.	86 585.3	{86 051., 92 859.}	10.6243	0.223824
23	newly 3 weeks spec	1235.	91.8308	{4., 258.}	92.5643	1.54391	newly 3 weeks cumul	1958.	156.908	{7., 452.}	91.9863	1.48819	newly 5 weeks spec	6598.	377.815	{14., 1006.}	94.2738	1.66387	newly 5 weeks cumul	11 490.	720.877	{28., 1986.}	93.726	1.62316	newly 65 weeks cumul	88 617.	87 426.1	{88 658., 93 097.}	8.54988	0.275942
24	newly 3 weeks spec	102.	2.77333	{0., 9.}	97.281	2.09555	newly 3 weeks cumul	138.	5.52	{0., 17.}	96.	1.90575	newly 5 weeks spec	2027.	4.98667	{0., 19.}	99.754	3.32266	newly 5 weeks cumul	2665.	14.3733	{0., 46.}	99.4607	2.83183	newly 67 weeks cumul	98 866.	32 762.7	{0., 78 436.}	66.8615	2.33147
25	newly 3 weeks spec	917.	15.6667	{0., 34.}	98.2915	2.41892	newly 3 weeks cumul	1410.	34.0556	{1., 85.}	97.5847	2.16413	newly 5 weeks spec	3941.	38.25	{0., 90.}	99.0294	2.86895	newly 5 weeks cumul	7263.	96.6111	{1., 208.}	98.6698	2.5492	newly 63 weeks cumul	94 839.	37 465.2	{1., 84 661.}	60.496	1.64216
26	newly 3 weeks spec	2138.	764.759	{36., 1683.}	64.3544	0.685043	newly 3 weeks cumul	3825.	1352.74	{77., 2922.}	64.9182	0.683767	newly 5 weeks spec	6408.	2526.26	{149., 5031.}	60.5765	0.611505	newly 5 weeks cumul	14 163.	5317.07	{300., 10 964.}	62.458	0.64233	newly 61 weeks cumul	87 857.	92 857.8	{92 576., 93 078.}	5.69197	0.0240409
27	newly 3 weeks spec	1999.	1565.19	{167., 3753.}	67.4531	0.486925	newly 3 weeks cumul	3536.	2998.95	{277., 7335.}	72.0447	0.487157	newly 5 weeks spec	6511.	3723.16	{539., 7565.}	51.1749	0.438027	newly 5 weeks cumul	13 890.	9233.66	{1097., 20 640.}	57.7746	0.458903	newly 61 weeks cumul	98 347.	87 658.6	{84 789., 90 023.}	10.868	0.0501053
28	newly 3 weeks spec	310.	6.9	{0., 15.}	97.7742	2.77092	newly 3 weeks cumul	474.	11.7	{0., 23.}	97.5316	2.66125	newly 5 weeks spec	2971.	21.38	{0., 77.}	99.2804	3.69575	newly 5 weeks cumul	4384.	44.78	{0., 129.}	98.9786	3.44812	newly 68 weeks cumul	89 828.	21 503.2	{0., 84 024.}	76.1817	3.82376
29	newly 3 weeks spec	609.	5.46512	{0., 18.}	99.1026	2.63125	newly 3 weeks cumul	951.	12.4186	{0., 31.}	98.6942	2.47321	newly 5 weeks spec	3642.	7.27907	{0., 19.}	99.8001	3.40891	newly 5 weeks cumul	6162.	25.6744	{0., 62.}	99.5833	3.06949	newly 65 weeks cumul	99 052.	28 070.4	{0., 67 568.}	71.6609	2.49364
30	newly 3 weeks spec	109.	1.92	{0., 6.}	98.2385	2.5161	newly 3 weeks cumul	138.	4.17	{0., 12.}	96.9783	2.23581	newly 5 weeks spec	1649.	6.53	{0., 19.}	99.604	3.64179	newly 5 weeks cumul	2255.	14.36	{0., 49.}	99.3632	3.30572	newly 68 weeks cumul	99 316.	15 122.6	{0., 74 938.}	84.7732	4.22203
31	newly 3 weeks spec	3137.	3369.15	{531., 6600.}	59.0771	0.315715	newly 3 weeks cumul	6051.	6898.32	{907., 13 855.}	66.3755	0.334092	newly 5 weeks spec	6713.	6248.33	{1529., 9682.}	33.9635	0.206768	newly 5 weeks cumul	17 548.	17 929.3	{3377., 31 984.}	48.5437	0.267454	newly 58 weeks cumul	87 424.	90 504.	{87 047., 92 361.}	3.89569	0.0165677
32	newly 3 weeks spec	1418.	278.946	{11., 675.}	80.8848	1.06657	newly 3 weeks cumul	2225.	485.324	{25., 1137.}	79.2189	1.01541	newly 5 weeks spec	5244.	1047.26	{71., 2504.}	80.0294	1.04566	newly 5 weeks cumul	10 289.	2083.	{130., 5008.}	79.9397	1.0502	newly 64 weeks cumul	98 867.	90 568.7	{88 767., 93 133.}	8.39344	0.112852
33	newly 3 weeks spec	501.	5.8	{0., 13.}	98.8423	3.06543	newly 3 weeks cumul	673.	11.08	{0., 28.}	98.3536	2.92151	newly 5 weeks spec	5187.	17.74	{0., 33.}	99.658	3.99782	newly 5 weeks cumul	7427.	38.83	{0., 79.}	99.4772	3.79454	newly 68 weeks cumul	99 003.	20 800.9	{0., 84 682.}	78.9896	4.02462
34	newly 3 weeks spec	1440.	218.619	{8., 575.}	84.8181	1.2442	newly 3 weeks cumul	2363.	385.952	{16., 1004.}	83.6668	1.22228	newly 5 weeks spec	6918.	810.984	{30., 2080.}	88.2772	1.37426	newly 5 weeks cumul	12 627.	1627.59	{58., 4205.}	87.1103	1.35654	newly 64 weeks cumul	95 674.	85 734.1	{86 355., 93 114.}	10.3893	0.397828
35	newly 3 weeks spec	179.	4.38	{0., 6.}	97.8883	2.65913	newly 3 weeks cumul	229.	8.38	{0., 11.}	96.9258	2.3647	newly 5 weeks spec	2514.	12.15	{0., 11.}	99.5167	3.72856	newly 5 weeks cumul	3425.	27.5	{0., 31.}	99.1971	3.33623	newly 68 weeks cumul	95 744.	13 739.7	{0., 72 452.}	85.6495	3.98704
36	newly 3 weeks spec	451.	3.29412	{0., 9.}	99.2696	2.50751	newly 3 weeks cumul	672.	8.	{1., 20.}	98.8095	2.21969	newly 5 weeks spec	3163.	4.76471	{0., 11.}	99.8494	3.38293	newly 5 weeks cumul	5077.	16.8627	{1., 38.}	99.6679	2.84515	newly 64 weeks cumul	99 501.	19 609.1	{2., 65 774.}	80.2926	1.91965
37	newly 3 weeks spec	142.	2.31373	{0., 4.}	98.3706	2.30222	newly 3 weeks cumul	189.	5.54902	{0., 12.}	97.064	1.86893	newly 5 weeks spec	2178.	4.94118	{0., 10.}	99.7731	3.37371	newly 5 weeks cumul	2952.	13.3922	{1., 24.}	99.5463	2.73429	newly 65 weeks cumul	99 424.	20 781.1	{1., 71 329.}	79.0985	2.40471
38	newly 3 weeks spec	1570.	502.207	{14., 1240.}	68.0123	0.813855	newly 3 weeks cumul	2591.	907.431	{29., 2198.}	65.0082	0.776809	newly 5 weeks spec	6266.	1603.57	{74., 3424.}	74.4084	0.902247	newly 5 weeks cumul	12 039.	3418.14	{122., 7891.}	71.6078	0.866686	newly 62 weeks cumul	93 543.	88 323.6	{86 063., 93 029.}	5.57966	0.120274
39	newly 3 weeks spec	955.	13.6346	{0., 22.}	98.5723	2.71361	newly 3 weeks cumul	1540.	28.75	{0., 52.}	98.1331	2.52153	newly 5 weeks spec	4980.	38.1731	{0., 56.}	99.2335	3.35596	newly 5 weeks cumul	8707.	89.2308	{0., 139.}	98.9752	3.01138	newly 64 weeks cumul	97 320.	34 438.7	{0., 76 927.}	64.6129	2.1187
40	newly 3 weeks spec	1152.	132.328	{1., 331.}	88.5132	1.60935	newly 3 weeks cumul	1821.	232.359	{5., 582.}	87.24	1.52683	newly 5 weeks spec	6088.	499.906	{0., 1332.}	91.7887	1.92924	newly 5 weeks cumul	10 814.	988.078	{7., 2663.}	90.863	1.80219	newly 65 weeks cumul	99 384.	72 569.3	{9., 93 074.}	26.9809	0.797454
41	newly 3 weeks spec	84.	1.68	{0., 5.}	98.	2.38647	newly 3 weeks cumul	118.	3.98	{0., 10.}	96.6271	2.11354	newly 5 weeks spec	1333.	3.39	{0., 12.}	99.7457	3.54105	newly 5 weeks cumul	1781.	9.62	{0., 25.}	99.4599	3.11017	newly 68 weeks cumul	96 330.	17 146.1	{0., 73 732.}	82.2007	3.81291
42	newly 3 weeks spec	2385.	2206.22	{378., 4599.}	60.0131	0.382375	newly 3 weeks cumul	4744.	4301.12	{638., 9189.}	61.6266	0.397861	newly 5 weeks spec	6151.	4826.88	{1155., 8333.}	41.2611	0.304916	newly 5 weeks cumul	14 873.	12 529.4	{2407., 23 961.}	50.3655	0.344983	newly 59 weeks cumul	96 441.	91 116.2	{89 422., 92 280.}	5.52134	0.0247512
43	newly 3 weeks spec	942.	447.547	{33., 908.}	63.2215	0.696142	newly 3 weeks cumul	1522.	789.141	{55., 1533.}	61.961	0.66017	newly 5 weeks spec	4281.	1546.33	{169., 3248.}	65.9013	0.759674	newly 5 weeks cumul	7799.	3185.08	{310., 6578.}	64.6988	0.731352	newly 64 weeks cumul	98 631.	90 717.6	{86 642., 93 054.}	8.0232	0.122787
44	newly 3 weeks spec	717.	2.4359	{0., 7.}	99.6603	2.85033	newly 3 weeks cumul	1097.	6.02564	{0., 12.}	99.4507	2.57621	newly 5 weeks spec	5449.	4.05128	{0., 9.}	99.9257	3.62904	newly 5 weeks cumul	8791.	12.6667	{0., 29.}	99.8559	3.21546	newly 64 weeks cumul	99 299.	19 576.9	{0., 67 460.}	80.2849	2.3455
45	newly 3 weeks spec	514.	1.83051	{0., 4.}	99.6439	3.09234	newly 3 weeks cumul	734.	4.54237	{0., 10.}	99.3811	2.65756	newly 5 weeks spec	4552.	4.86441	{0., 9.}	99.8931	3.93038	newly 5 weeks cumul	6924.	12.5085	{0., 23.}	99.8193	3.40115	newly 66 weeks cumul	99 357.	16 932.3	{0., 67 651.}	82.9581	3.20678
46	newly 3 weeks spec	247.	5.6	{0., 14.}	97.7328	2.74134	newly 3 weeks cumul	335.	10.77	{0., 28.}	96.7851	2.6893	newly 5 weeks spec	2717.	20.28	{0., 29.}	99.2536	3.65673	newly 5 weeks cumul	3855.	41.72	{0., 69.}	98.9178	3.56157	newly 68 weeks cumul	99 095.	19 842.3	{0., 78 509.}	79.9765	4.02828
47	newly 3 weeks spec	977.	213.667	{11., 439.}	78.604	0.991498	newly 3 weeks cumul	1597.	405.039	{27., 804.}	76.6118	0.928673	newly 5 weeks spec	4864.	622.706	{23., 1166.}	87.1977	1.23928	newly 5 weeks cumul	8755.	1392.9	{74., 2689.}	84.0902	1.14063	newly 63 weeks cumul	96 045.	82 577.5	{84 299., 85 110.}	14.0221	0.173547
48	newly 3 weeks spec	364.	3.95313	{0., 10.}	98.914	2.58551	newly 3 weeks cumul	527.	8.17188	{0., 17.}	98.4494	2.34086	newly 5 weeks spec	3183.	6.10938	{0., 14.}	99.8081	3.45907	newly 5 weeks cumul	4879.	19.	{0., 36.}	99.6106	3.02015	newly 66 weeks cumul	97 052.	27 416.7	{0., 67 669.}	71.7505	2.31148
49	newly 3 weeks spec	1900.	2394.89	{312., 5299.}	80.0965	0.375065	newly 3 weeks cumul	3162.	4037.81	{504., 9032.}	83.0505	0.383995	newly 5 weeks spec	7611.	7203.75	{1649., 12 364.}	43.0674	0.263576	newly 5 weeks cumul	14 764.	15 739.8	{2829., 30 584.}	57.4134	0.311118	newly 64 weeks cumul	95 259.	92 513.7	{88 294., 94 005.}	2.88191	0.0129674
50	newly 3 weeks spec	943.	137.981	{2., 385.}	85.3679	1.3543	newly 3 weeks cumul	1533.	239.385	{4., 672.}	84.3846	1.26735	newly 5 weeks spec	5024.	555.212	{2., 1435.}	88.9488	1.60184	newly 5 weeks cumul	8966.	1077.4	{6., 2897.}	87.9835	1.49237	newly 65 weeks cumul	98 082.	78 092.	{34 918., 93 121.}	20.3809	0.567812
